# Azithromycin-carrying and microtubule-orientated biomimetic poly (lactic-co-glycolic acid) scaffolds for eyelid reconstruction

**DOI:** 10.3389/fmed.2023.1129606

**Published:** 2023-05-16

**Authors:** Peifang Xu, Pengjie Chen, Qi Gao, Yiming Sun, Jing Cao, Han Wu, Juan Ye

**Affiliations:** Eye Center, The Second Affiliated Hospital, School of Medicine, Zhejiang University, Zhejiang Provincial Key Laboratory of Ophthalmology, Zhejiang Provincial Clinical Research Center for Eye Diseases, Zhejiang Provincial Engineering Institute on Eye Diseases, Hangzhou, Zhejiang, China

**Keywords:** eyelid reconstruction, biomimetic, tarsal plate substitute, oriented structure, poly(lactic-co-glycolic acid)

## Abstract

**Introduction:**

Tarsal plate repair is the major challenge of eyelid reconstruction for the oculoplastic surgeon. The ideal synthetic tarsal plate substitute should imitate the microstructure and mechanical strength of the natural eyelid. The aim of this work was to develop a novel bionic substitute for eyelid reconstruction.

**Methods:**

Three types of poly(lactic-co-glycolic acid) (PLGA) scaffolds (random, oriented, and azithromycin-loaded oriented scaffolds) were prepared using an improved thermal-induced phase separation technique. The microstructure of the scaffolds was examined by scanning electron microscopy. In vitro cytotoxicity was assessed using scaffold extracts. Fibroblast and primary rat meibomian gland epithelial cells (rMGCs) were cultured within the scaffolds, and their behavior was observed using fluorescence staining. Three types of PLGA scaffolds were implanted into rabbit eyelid defect in vivo to evaluate their inductive tissue repair function.

**Results:**

We successfully fabricated three types of PLGA scaffolds with varying pore architectures, and the axially aligned scaffold demonstrated interconnected and vertically parallel channels. In vitro cytotoxicity tests using scaffold extracts revealed no apparent cytotoxicity. Fluorescence staining showed that both Fibroblast and rMGCs could adhere well onto the pore walls, with fibroblast elongating along the axially aligned porous structure. At 8 weeks post-implantation, all scaffolds were well integrated by fibrovascular tissue. The axially aligned scaffold groups exhibited faster degradation compared to the random scaffold group, with smaller fragments surrounded by mature collagen fibers.

**Conclusion:**

The study found that the axially aligned scaffolds could well support and guide cellular activities in vitro and in vivo. Moreover, the axially aligned scaffold group showed a faster degradation rate with a matched integration rate compared to the random scaffold group. The findings suggest that the oriented scaffold is a promising alternative for eyelid tarsal plate substitutes.

## 1. Introduction

Eyelids is an important functional structure of eye, which can provide corneal protection and offer good cosmesis (1). Eyelid defects usually result from congenital anomalies, trauma, tumor excision, burns, or autoimmune disease (2). The goal of eyelid reconstruction is to restore structural and functional integrity of the eyelid. The posterior lamella is the key of eyelid reconstruction, which can provide structural support and a mucosal surface that allows for corneal protection. To reconstruct this layer, various tarsal substitute materials have been developed. Homologous grafts such as contralateral eyelid (3), hard-plate mucosa (4), nasal or ear cartilage grafts (5) were most often used. However, surgery is needed to harvest the homologous grafts, which may cause additional injury to the donor site. Xenogenic substitutes such as allogeneic sclera, acellular dermal matrix (ADM) (6), and scleral patches (7) were restricted in application for infection and immunologic rejection. Therefore, synthetic tarsal substitute may be an effective solution to alleviate donor shortage, immune rejection and infection risks.

An ideal scaffold for tarsal substitute should exhibit similar structural and mechanical properties to the targeted tissue (8). Previous studies have revealed the effect of pore size and geometry in promoting tissue regeneration using porous scaffolds with radially aligned or axially aligned pores. The scaffolds with regular pores were designed to better mimic the microenvironments of the target tissues, which possess oriented structure. Xu, et al. designed a scaffold with microstructure orientation for cartilage tissue engineering. By optimizing the freezing box and temperature, the manufacture PLGA scaffold has a mechanical strength and microstructure close to that of real cartilage (9). Dai. et al. applied the oriented PLGA scaffolds into rabbit articular osteochondral defect models, and the results confirmed an *in situ* inductive osteochondral regeneration without pre-seeding cells (10). Tarsal plate is very superficial, and located between two very thin tissues, the conjunctiva and eyelid skin. Too hard or non-degradable materials, such as Medpor, may cause cornea irritation, exposure or thick fibrous encapsule in long term observation (11). So far, few researches have tried degradable or materials with oriented microstructure to eyelid reconstruction. Therefore, oriented PLGA scaffolds may be a promising material for eyelid repair for its regular structure and similar mechanical strength with cartilage (12).

Azithromycin (AZM) is a potent broad-spectrum macrolide antibiotic. Studies have proved the potentials of AZM for localized therapy, which enables the delivery of antibiotic to the site of action at lower doses while escaping systemic drug effects and reducing the risk of developing microbial resistance (13, 14). What’s more, AZM was available at the surgical site for a longer period of time than amoxicillin, and patients taking azithromycin exhibited lower levels of specific proinflammatory cytokines (15). As for eyelid, AZM acts directly on meibomian gland epithelial cells to stimulate their differentiation, enhance the quality and quantity of their lipid production, and promote their holocrine secretion (16). Thus, AZM may enhance resolution of postoperative inflammation and enhance the local meibomian gland function, simultaneously.

The aim of this work was the development of a bionic tarsal plate substitute. By using ice particulate templates and freeze-drying, an AZM-carrying and microtubule-orientated PLGA scaffolds can be prepared successfully. The behaviors of two target cells of tarsal plate in the PLGA scaffolds were evaluated. Moreover, we intend to investigate the effects of the different pore architectures of the three scaffolds on the repair of eyelid defects *in vivo*. It is expected that this study will contribute towards the development of a novel substitute for eyelid reconstruction.

## 2. Materials and methods

### 2.1. Materials

PLGA (molar ratio of lactyl/glycotyl = 75/25, Mw = 1.22kd) was purchased from China Textile Academy. 1,4-Dioxane, ethanol, and other reagents of analytical grade were directly used in this study. Dulbecco’s modified Eagle’s medium (DMEM, Gibco, United States) and fetal bovine serum (FBS, Gibco, United States) were used for *in vitro* cell culture.

### 2.2. Preparation of PLGA scaffolds

The PLGA scaffolds were prepared by ice-templated assembly and temperature gradient-guided thermally-induced phase separation, which controlled the direction of crystal growth (17). The mold structures used to fabricate the random, axially aligned PLGA scaffolds are shown in Supplementary Figure S1. In brief, 5% w/v PLGA in dioxane solution, and PE and PTFE molds were preheated at 37.0°C for 1 h. To prepare the random PLGA scaffold, the PLGA solution in the PE mold was transferred into a freezer at −20°C overnight to form randomly distributed crystals. To fabricate the axially aligned scaffold, PE molds containing the PLGA solution were mounted into the holes of a larger PTFE mold, which was placed between two −20°C precooled aluminum molds. The system was maintained at −20°C overnight to ensure temperature transduction in an axially directional manner. After the PLGA solution was absolutely frozen, it was lyophilized to remove dioxane solution and preserve the formed porous structure. 1 mg Azithromycin was dissolved into 400 μL 5% w/v PLGA solution. The mixture solution was used to fabricate the Azithromycin-carrying, axially aligned scaffold at the same method. Finally, three types of scaffolds were fabricated with different pore architectures, namely R-PLGA, O-PLGA, and AZM@O-PLGA. R-PLGA refers to PLGA scaffolds with random pores. O-PLGA refers to PLGA scaffolds with axially aligned pores. AZM@O-PLGA refers to oriented PLGA scaffolds loaded with AZM.

### 2.3. Characterization of PLGA scaffolds

The microstructure of three types of PLGA scaffolds was characterized using a field emission scanning electron microscope (FESEM; S4800, Hitachi, Japan). The Azithromycin loading efficacy was determined by color reaction of sulfuric acid. Firstly, AZM@O-PLGA scaffolds were prepared according to the description of section 2.2. Secondly, an Azim solution standard curve was plotted Supplementary Figure S2. To this end, different concentrations of AZM (0, 7.5, 10, 15, 20, 25, and 30 μg/mL) were prepared and the standard curve was then established based on the relationship between the azithromycin standard solution and the corresponding absorbance at 482 nm. To measure the encapsulation efficiency of AZM on the PLGA scaffolds, each of the AZM loaded samples were placed in a 1.5 mL Eppendorf tube. Then, 500 mL of ethanol was added to dissolve the AZM in the samples. Afterwards, the supernatant was collected by centrifugation at 8000 rpm for 5 min. To determine the content of AZM in each sample, the absorbance of supernatant at 482 nm was measured and the AZM loading efficacy was calculated using the following equation:


$$\mathrm{AZM}\;\mathrm{loading efficacy}=W_{r}/W_{i}$$

where W_i_ is the initial added amount of AZM and W_r_ is the remaining amount of AZM encapsulated in the samples.

### 2.4. Cell culture

Human dermal fibroblasts (HDFs, kindly provided by Faculty of Burn, Second Affiliated Hospital of Zhejiang University, Hangzhou, China). HDFs were cultured in DMEM, supplemented with 10% FBS and penicillin/streptomycin (100/100 U) at 37°C and 5% CO2. The primary rat meibomian gland epithelial cells (rMGCs) were isolated according to the previous study (18). In brief, the eyelid tissues were obtained freshly from ~120 g Sprague–Dawley (SD) rats. The tarsal plates were isolated from eyelid tissues under a dissecting microscope by removing skin, subcutaneous tissue, muscle, and palpebral conjunctiva. Small pieces of tarsal plates, which contained two to five meibomian glands, were further digested with 0.25% collagenase I (Biosharp, China) and 0.9 U/mL dispase II (Roche Applied Science, Indianapolis, IN) at 37°C for 5 h. Single glands were then isolated under a dissecting microscope and were dissociated into a single-cell suspension by 0.05% trypsin- EDTA treatment for 5 min. Isolated cells were centrifuged at 1000 rpm for 5 min resuspended and cultured in keratinocyte serum-free medium (SFM) (Invitrogen-Gibco, United States) for 5 to 7 days before subculture. To detect the neutral lipid expression, the cells were stained with 0.3% Oil red O (Beyotime Biotechnology, China) and lipophilic dye Nile red (MedChemExpresss, United States).

### 2.5. RNA extraction and RT-PCR

The gene expression of keratin 14 (Krt 14), keratin 5 (Krt 5), p63, Sox9 and PPAR- 𝛾 in rMGCs was identified by RT-PCR. Total RNA of cells was extracted using FlaPure Animal Tissue/Cell DNA Extraction Kit (Genesand Biotech, China) according to the manufacturer’s protocol. The RNA was reverse transcribed into cDNA using a PrimeScript RT Reagent Kit (Takara, China), and the resulting cDNA was utilized for PCR amplification. Real-time PCR (RT-PCR) reactions were conducted using the SYBR Premix EX TaqTM kit (Takara, China) and the CFX96 system (Bio-Rad, United States), with the reaction conditions of 95°C for 30 s, followed by 40 cycles of 95°C for 5 s and 60°C for 30 s. The expression of β-actin was used as a reference gene to normalize the real-time PCR results, and the relative mRNA expression was calculated using the comparative cycle threshold method (ΔΔCT method). The primer sequences utilized in RT-PCR studies are provided in Supplementary Table S1. All the experiments were conducted in triplicate.

### 2.6. *In vitro* cytotoxicity

To evaluate the potential cytotoxicity of scaffolds, three scaffolds were first immersed in DMEM or SFM at an approximate concentration of 0.1 g/mL and incubated at 37°C for 24 h to obtain the scaffold extraction solutions. The extracted solutions were then collected and was filtered by a 0.22 μm bacteria-retentive filter. The HDFs and rMGCs were seeded into a 96-well plate at a density of 5 × 10^3^ and 8 × 10^3^ cells per well, respectively. After 24 h of incubation, the medium was removed and replaced with the extracts with the same supplements as negative control(fresh medium with no extracts). After another 24 h continuous cultivation, 100 μL cell culture medium supplemented with 10 μL CCK-8 solution(Dojindo Laboratories, China) was added to each well. After 2 h incubation at 37°C, the absorbance at 450 nm was assessed using a SpectraMax iD5 microplate reader (Molecular Devices, United States).

### 2.7. Cell behaviors *in vitro*

The distribution and morphology of HDFs and rMGCs in the PLGA scaffolds were visualized using an inverted fluorescence microscope (Leica, German) and a confocal laser scanning micro- scope (CLSM; LSM780, ZEISS, Germany). HDFs and rMGCs with a concentration of 5 × 10^6^ mL^−1^ were introduced into the PLGA composite scaffolds under negative pressure, respectively. Then, the cell seeded scaffolds were maintained at 37°C and 5% CO_2_ for 3 h to allow initial adhesion of onto the scaffolds. The complete DMEM was then added to the HDFs seeded scaffolds, and keratinocyte SFM to the rMGCs seeded scaffolds. Following 2 days of cultivation, cell viability was assessed using the Live/Dead assay. Specifically, the PLGA scaffolds were incubated with calcein-AM/propidium iodide (PI; Beyotime Biotechnology, China) for 30 min at 37°C and evaluated using a fluorescence microscope. After 5 days cultivation for HDFs and 7 days for rMGCs, the PLGA scaffolds were fixed with 4% w/v paraformaldehyde at 37°C for 1 h. The cells were permeabilized in 0.1% w/v Triton X-100 at 4°C for 10 min, followed by blocking with 1% BSA at 37°C for 1 h. The nucleus and cytoskeleton of HDFs were stained with Hoechst 33342 and FITC-labeled phalloidin at 4°C overnight for fluorescence microscope observation. The nucleus and cytoskeleton of rMGCs were stained with DAPI and rhodamine phalloidin at 4°C overnight for CLSM observation.

### 2.8. *In vivo* rabbit eyelid defect model and scaffolds transplantation

Three groups of PLGA scaffolds were cut into pieces at an average size of 8.0 × 4.0 mm, which were sterilized in 75% ethanol for 4 h, and then displaced with sterile normal saline before use.

All animal procedures were performed in accordance with the Guidelines for Care and Use of Laboratory Animals of Hangzhou Medical College and approved by the Animal Ethics Committee of Hangzhou Medical College. Twelve male New Zealand white rabbits weighing between 2.5 and 3.0 kg were divided into three groups (4 eyes per group). The rabbit eyelid defect model was stablished according to our previous study (19). In brief, the rabbits were anesthetized by an intravenous injection of pentobarbital sodium (30 mg kg^−1^), followed by local anesthesia with 1% lidocaine. The lower eyelid was everted to expose the palpebral conjunctiva. An 8.0 × 4.0 mm defect was created inside the lid with a micro scissor to completely remove the conjunctival epithelium and substantia propria. PLGA scaffold was grafted to the wound bed by interrupted sutures using 7–0 polyglactin. The usage of ofloxacin ointment in the conjunctival sac was performed after surgery. At the end of 1 and 2 month, the morphology of repaired eyelid was observed. All rabbits were euthanized at 2 months after surgery to harvest the eyelid tissues containing the defect and normal area for further histological analysis.

### 2.9. Statistical analysis

GraphPad Prism 8.0 Software (GraphPad Software Inc., United States) was used to analyze the data. The differences between groups were examined with One-way ANOVA. A value of *p* < 0.05 was considered statistically significant.

## 3. Results

In this study, three types of scaffolds, i.e., R-PLGA, O-PLGA, and AZM@O-PLGA were prepared, and their ability to repair eyelid defects were compared *in vivo*.

### 3.1. Microstructure of three types of PLGA scaffolds

**Figure 1:**
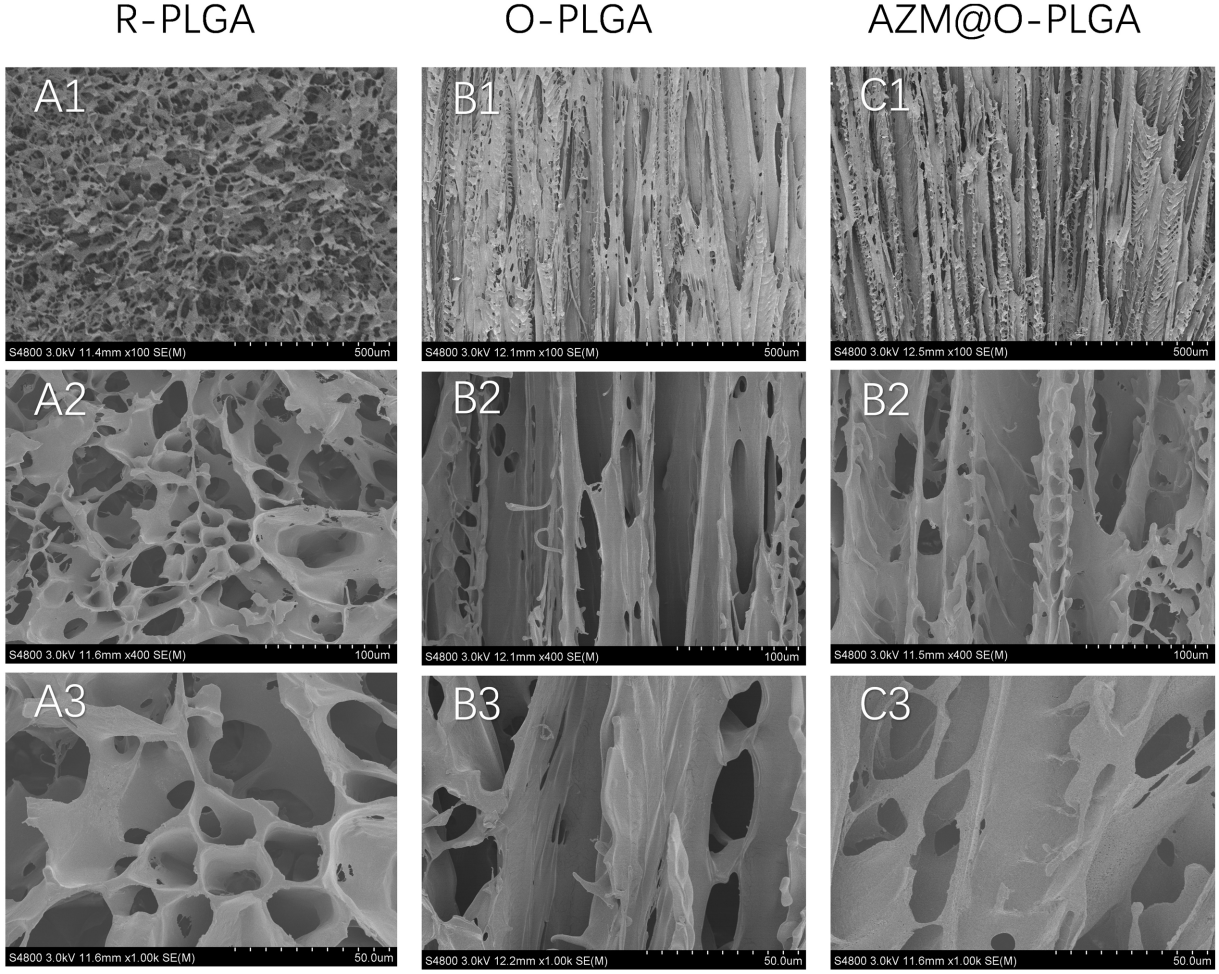
SEM images showing the morphology of random structured **(A1–A3)**, and orientation-structured PLGA scaffolds **(B,C)**. Azithromycin loading **(C1–C3)** did not influent the orientated structure.

The methods used to prepare the three types of PLGA scaffolds are shown in Supplementary Figure S1. The random scaffold was obtained by phase-separation at low temperature of −20°C, whereas the axially aligned scaffolds were prepared by thermally induced phase separation under an axial temperature gradient, followed by lyophilization. The morphology of the three kinds of PLGA scaffolds is shown in Figures 1A–C, confirming the desired types of architectures. The random scaffold exhibited an evenly irregular porous structure in the vertical section (Figures 1A1–A3). The axially aligned scaffold showed interconnected and vertically parallel channels (Figures 1B1,B2,C1–C3), validating the good efficacy of preparation of the axially aligned scaffolds by this method. The average AZM loading efficacy is 84.86 ± 6.15%. There was no significant difference in the architectures of the axially aligned scaffold with or without AZM loading.

### 3.2. Cultivation and identification of primary rat meibomian gland epithelial cells (rMGCs)

**Figure 2:**
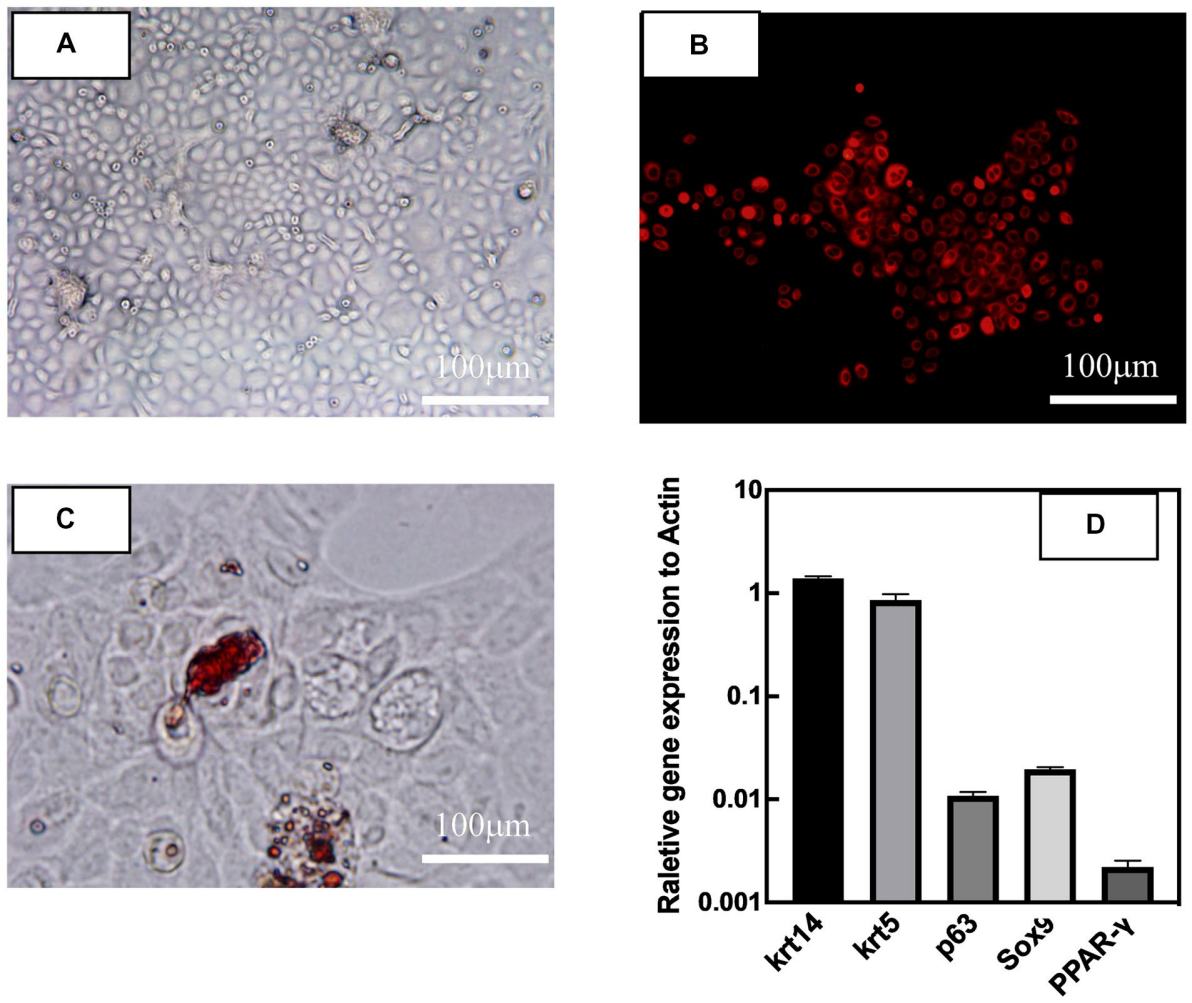
Appearance of primary rat meibomian gland epithelial cells (passage 2) after culturing in SFM for 5 days. Light microscopy image **(A)**, Nile red staining **(B)**, oil O staining**(C)** of meibomian gland epithelial cells, respectively. Gene expression patterns **(D)** of rat meibomian gland epithelial cells. Data are shown as mean ± SD and each sample was tested in triplicate.

The primary rMGCs had cobblestone morphology day 5 days (Figure 2A). Neutral lipid droplets in primary human meibomian gland epithelial cells were detected by Nile red and Oil red O staining. Nile red staining (Figure 2B) was predominantly localized to the cell membrane and showed weak intracellular staining, indicating a lower presence of lipid droplets in the cells cultured in SFM. Additionally, the results of Oil red O staining (Figure 2C) showed that there was minimal intracellular lipid staining in SFM-cultured cells. The phenotype of rMGCs (Figure 2D) was identified by RT-qPCR with positive expression of keratin-related genes krt 14 and krt 5. Very low expression of lipid-related gene PPAR-γ, as well as positive expression of stem cell marker sox9 and p63 showed that the harvested cell could proliferate well *in vitro*, maintaining the proliferation characteristic of progenitor cells.

### 3.3. *In vitro* cytotoxicity of PLGA scaffolds

**Figure 3:**
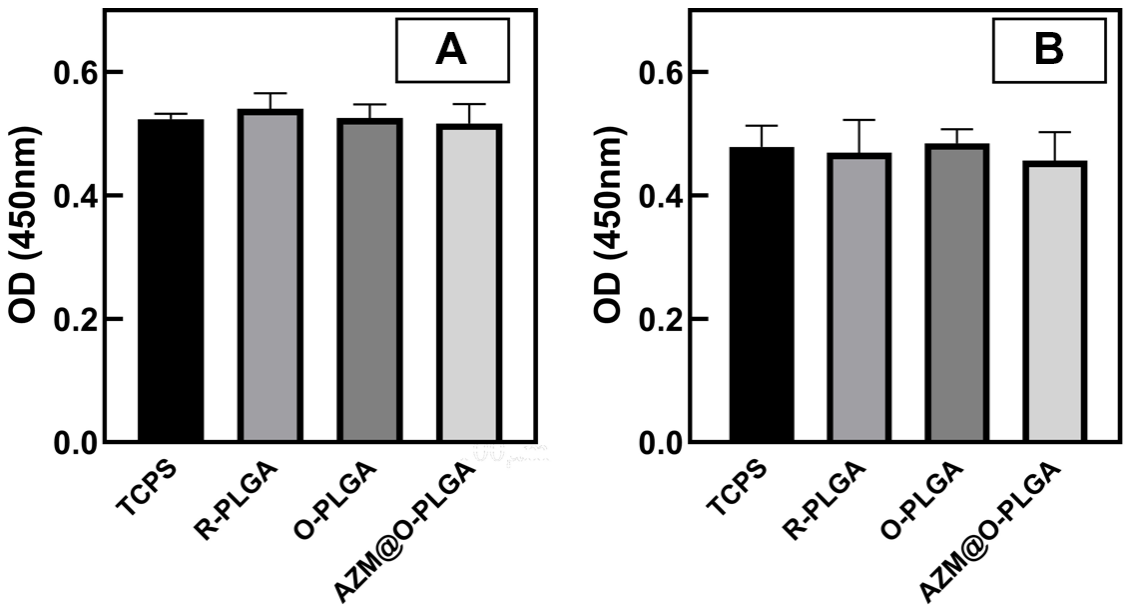
Cytotoxicity of the scaffolds. CCK-8 assay of human fibroblast cells **(A)** and rat meibomian gland epithelial cells **(B)** cultured with extracts of scaffolds. Data are shown as mean ± SD and each sample was tested in triplicate.

The cytotoxicity of the three scaffolds was assessed through the CCK-8 assay. The extracted solutions of the scaffolds were incubated with HCFs and rMGCs, and the OD values were measured and compared to normal medium. The extraction solutions of the three scaffolds did not induce significant changes in cell viability of HDFs (Figure 3A) and rMGCs (Figure 3B).

### 3.4. Cell behavior of HDFs and rMGCs on PLGA scaffolds

**Figure 4:**
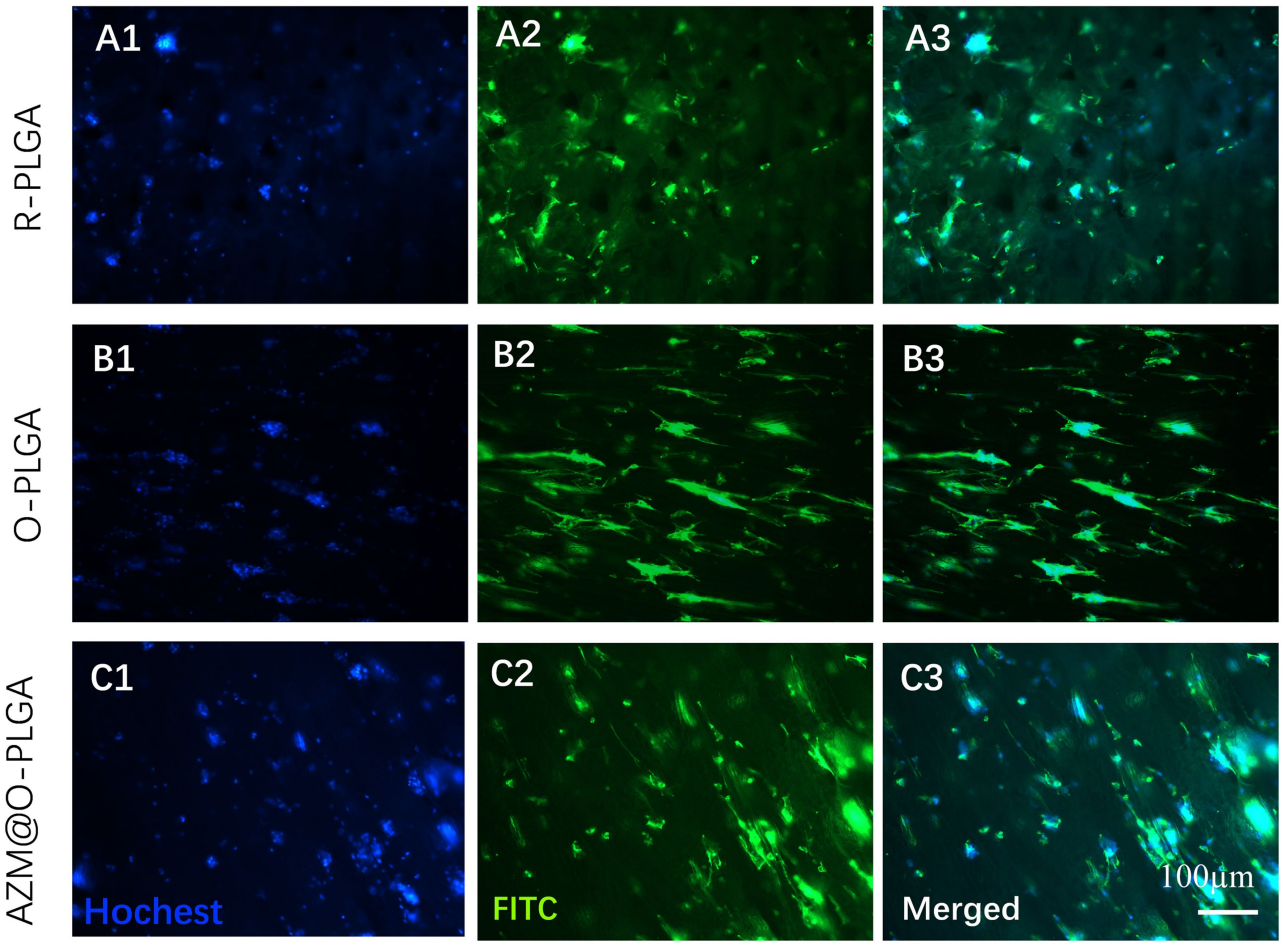
Fluorescence staining showing the morphology and distribution of human fibroblast cells inside **(A)** R-PLGA, **(B)** O-PLGA and **(C)** AZM@O-PLGA scaffolds after 5 days culture *in vitro*. The nuclei and actin were stained with hochest-33342 (blue) and FITC phalloidin (green), respectively..

**Figure 5:**
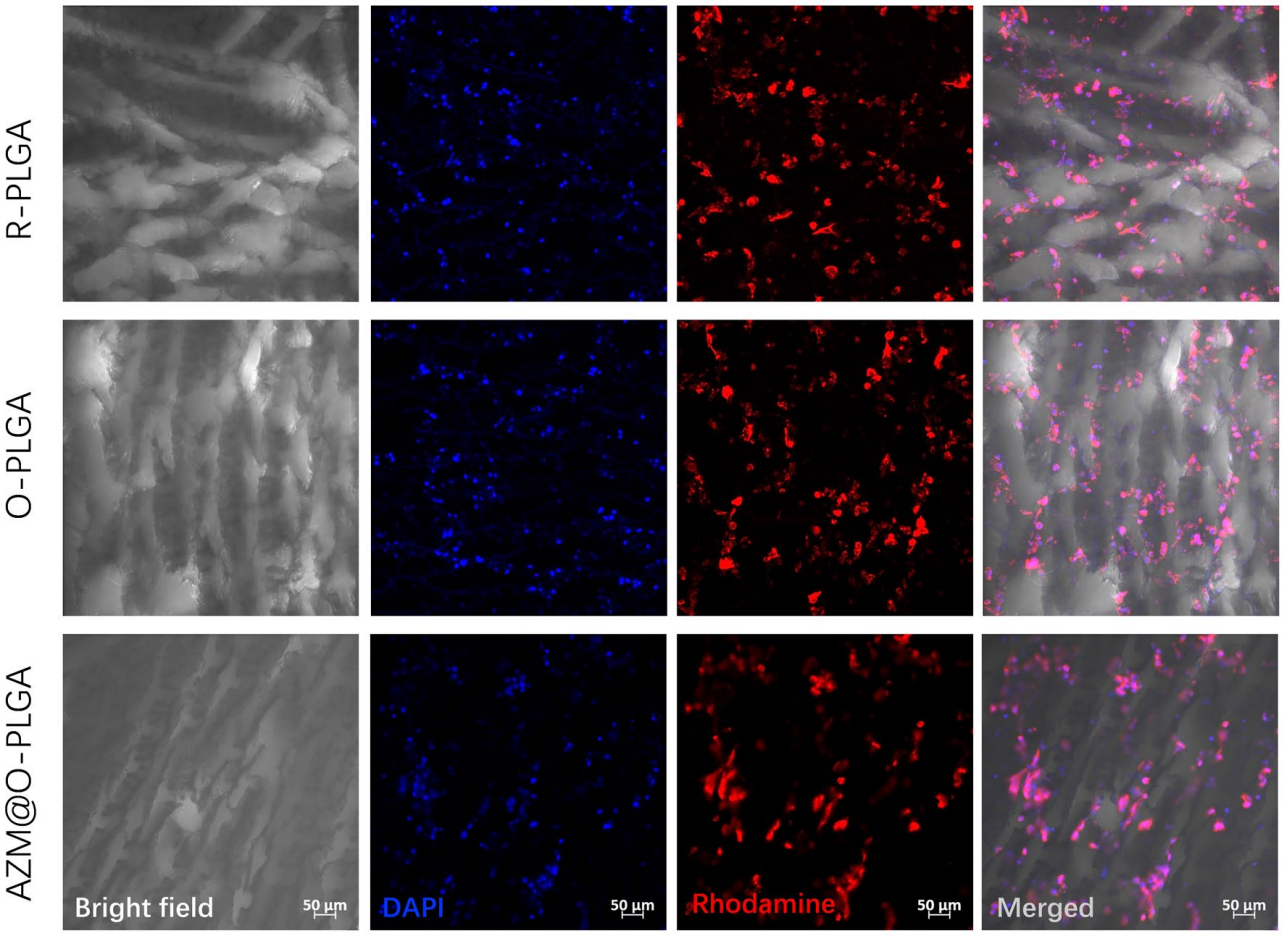
CLSM images showing the morphology and rat meibomian gland epithelial cells inside three scaffolds after 7d three-dimension culture *in vitro*. The nuclei and actin were stained with DAPI (blue) and rhodamine phalloidin (red), respectively.

The distribution and morphology of HDFs and rMGCs in the scaffolds were observed by an inverted fluorescence microscope (Leica, Japan) and a confocal laser scanning microscopy (CLSM; LSM780, ZEISS, Germany), respectively. Fluorescence microscope observation revealed that HDFs could adhere and spread onto the three PLGA scaffolds (Figure 4) with a high degree of viability, as confirmed by Live/dead assay (Supplementary Figure S3) which showed minimal cell death. Moreover, the HDFs distributed randomly on the random PLGA scaffold (Figures 4A1–A3), while they were spread completely and elongated along the axially aligned porous structure on the O-PLGA (Figures 4B1–B3), and AZM@O-PLGA (Figures 4C1–C3) scaffolds. Similarly, Live/dead assay indicated that nearly all rMGCs on the three scaffolds were alive (Supplementary Figure S4). The rMGCs were uniformly distributed in the three types of PLGA scaffolds, after 7 days cultivation. CLSM observation showed the rMGCs could adhere well onto the pore walls (Figure 5).

### 3.5. Reconstruction of eyelid defect by PLGA scaffolds.

**Figure 6:**
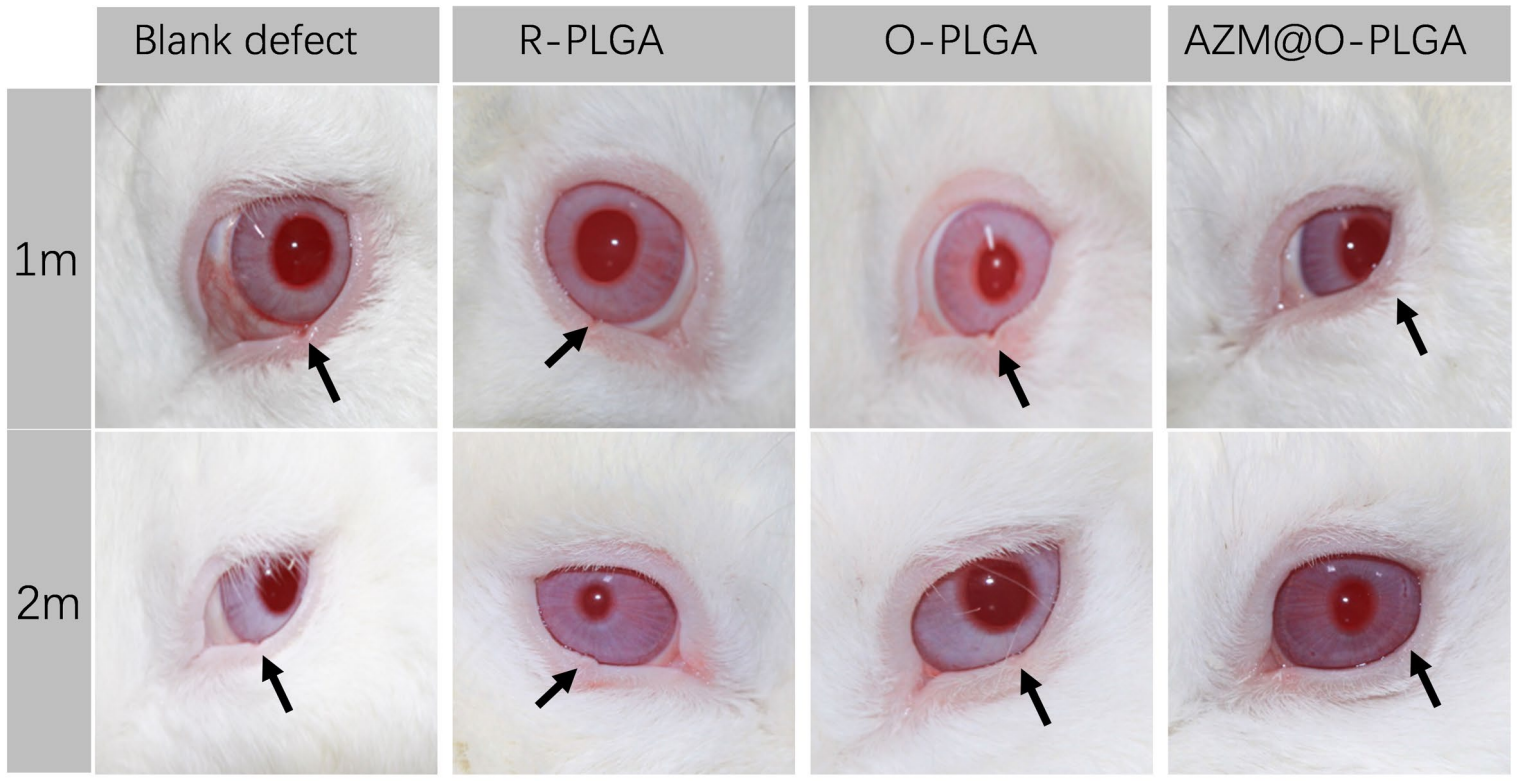
Post-surgery appearance of repaired eyelid after the full thickness defects (6.0 × 3.0 mm) were reconstructed by R-PLGA, O-PLGA and AZM@O-PLGA scaffolds at 1 and 2 months. The black arrows point to the defect sites.

**Figure 7:**
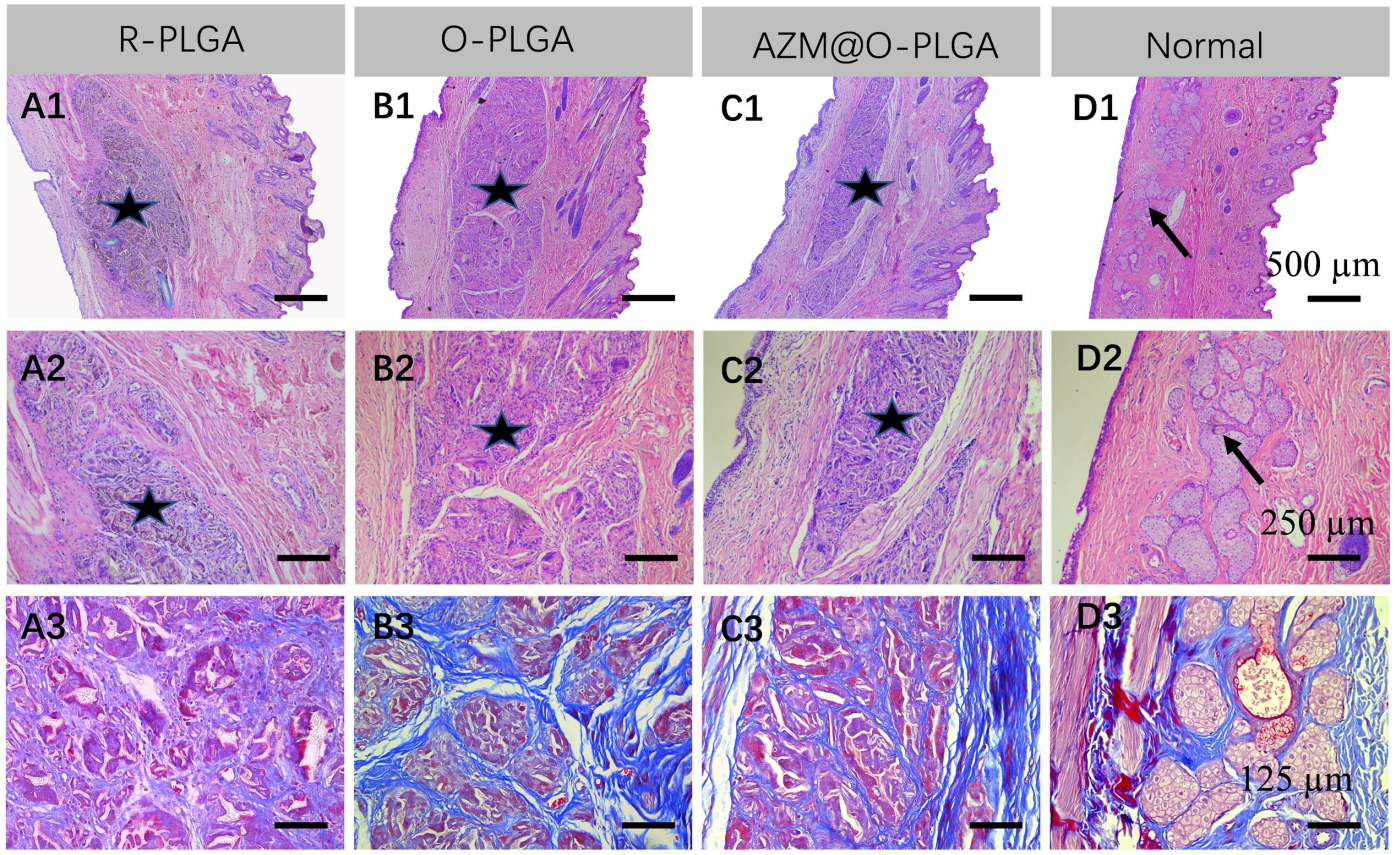
Histological analysis of the repaired eyelid implanted by **(A)** R-PLGA, **(B)** O-PLGA and **(C)** AZM@O-PLGA scaffolds for 8 w, respectively. H&E staining **(A1–D1,A2-D2)** and Masson staining **(A3-D3)** of the repaired and normal eyelid tissues. Asterisks represent scaffolds. Black arrows point to the normal meibomian glands.

As three types of PLGA scaffolds would well support both HDFs and rMGCs, we further applied the scaffolds as grafts to reconstruct the eyelid defect in a rabbit model. Rabbits in all groups ate normally and behaved regularly in the weeks after operation. Though at 1 m of post-operation, the eyelid grafted with R-PLGA and O-PLGA scaffolds formed a noticeable notch at the wound site. All grafted eyelid showed acceptable defect repair with mild scar at 2 m of post-operation (Figure 6). The eyelid grafted with AZM@O-PLGA scaffolds showed good wound healing without noticeable scar formation or other abnormality. Historically, at 2 m of post-operation, all PLGA scaffolds started to degrade into pieces and were well integrated by fibrovascular tissue. The R-PLGA degraded slower than O-PLGA scaffolds, with more PLGA material and less collagen deposition inside scaffolds (Figures 7A1–A3; Supplementary Figures S5A1,A2; Supplementary Figures S6A1,A2). The O-PLGA scaffolds and AZM@O-PLGA scaffolds were markedly degraded into small fragments surrounded by mature collagen fibers (Figures 7B1–C3; Supplementary Figures S5B1–C2; Supplementary Figures S6B1–C2), simulating the structure of natural tarsal plate (Figures 7D1–D3; Supplementary Figures S6D1,D2), while the untreated defected tissue showed a denser collagen deposition (Supplementary Figures S6D1–E2).

## 4. Discussion

Tarsal plate is an essential structure that support and maintain shape of eyelid. The structure of tarsus is composed of regularly arranged meibomian glands and surrounding ECM. Because of the particularity of tissue component and regularity of tissue distribution, there is a lack of suitable eyelid tarsus tissue substitutes, making it a big challenge in eyelid defect reconstruction. In this study we tried to construct an axially aligned PLGA scaffold which is structurally similar to the native tarsus ECM. *In vitro* study showed that the O-PLGA scaffolds are able to support the growth of the two target cells, fibroblasts and meibomian glands cells. In addition, after transplanted into the defect site of eyelid, the O-PLGA, especially AZM@O-PLGA could well maintain the normal shape of eyelid with mild scare formation, and be well integrated by the local tarsus tissue.

Many native tissues had oriented structures, such as tendon, cartilage, nerve, bone and so on. The oriented structures are closely related to their physiological and mechanical properties (10). Scaffolds were fabricated with oriented structures to mimic the microenvironment of native tissue, and were proved to facilitate cell promotion, differentiation, spatial organization and tissue formation (20). To a certain degree, tarsus has its own oriented structure caused by axially arrangements of meibomian glands. We conjectured that oriented scaffolds may induce eyelid ECM remodeling. To enable injured eyelid tissue to recover its normal function and promote tissue repair, tissue engineering eyelid tarsus tissue substitutes should mimic native tissue, both mechanically and physiologically. Therefore, we fabricated three types of PLGA scaffolds for eyelid defect reconstruction. The axially aligned PLGA was designed to mimic the natural structure of eyelid tarsus tissue.

Some researchers have attempted to develop a synthetic tarsal substitute for eyelid defect reconstruction caused by tumor resection, trauma, or congenital diseases. Synthesized materials such as porous HDPE (Medpor), poly(3-hydroxybutyrate-co-3-hydroxyhexanoate) (21), and poly(propylene fumarate)-2-hydroxyethyl methacrylate copolymers (22) have been explored as tarsus substitutes. However, these synthesized materials were rigid and hardly degradable. Long-term implantation may lead to complications, such as exposure through the skin, unexplained pain, poor mobility and eyelid abnormal shape (11). Very few studies tried degradable biomaterials for eyelid reconstruction. Michelle T. Sunat al. developed a macro-porous chitosan scaffold for eyelid tarsus tissue engineering, which could support the attachment and proliferation human orbital skin fibroblasts *in vitro* (23). But the authors did not investigate the scaffolds for further *in-vivo* repair effects.

In clinical, a lid-sharing technique was wildly used for large eyelid defects. The shared lid is usually divided about 2 months after the initial surgical repair to allow enough time for the reconstructed lid to develop a new blood supply and to counteract the downward contractile forces of scar maturation and gravity (24). Therefore, scaffolds should have a good balance between degradation rate and tarsus tissue repair after implanted in defect sites, except for good biocompatibility and mechanical intensity. PLGA are the most popular polymers in tissue engineering, such as bone (25), tendon (26), cartilage (27) and so on, due to their biodegradability and biocompatibility. Oriented PLGA scaffolds have shown great potential for the practical application in cartilage regeneration, for biomimetic structure and mechanical property (28). Based on the axially aligned structure of tarsus tissue and specific mechanical property similar to cartilage (12), we suspected that the multiple-bionic oriented PLGA scaffolds may have great potential in eyelid defect repair. After 2-month *in vivo* observation, the implanted oriented PLGA substitute was well degraded into fragments and surrounded with collagenous fiber, which was similar with natural eyelid in morphological character (Figure 6). The relatively slower degradation of R-PLGA substitute may due to pore structure and interconnectivity. Preliminary observation showed a matched rate between oriented PLGA degradation and eyelid tissue integration.

As foreign materials that have been introduced into the body, biomaterials would also be a potential source of infection (29) and associated with triggering inflammation and immune reactions (30). Therefore, multifunctional biomaterials with combined properties that can combat infections, modulate inflammation, and promote regeneration is the real quest. Azithromycin antibiotics have been reported to have anti-inflammatory properties in blepharitis and meibomian gland dysfunction, by suppressing the expression of proinflammatory mediator, such as IL-1β, IL-8, and MMP-9 (31). In this study, AZM was loaded into the microtubule-orientated biomimetic PLGA scaffolds. This biomaterial was developed not only as a substitute of eyelid tarsus, but also as a mediator to prevent biodevices-related infections and improve the local environment of eyelid defect. Preliminary results of animal experiments showed that AZM@O-PLGA scaffolds have a batter eyelid repair effects after 1 month and 2 months transplantation. However, this article is only a first attempt to evaluate the *in vivo* repair effect of oriented scaffolds loaded with AZM as eyelid tarsus substitutes. In the future study, longer observation *in vivo* and more functional characterization of the two target cells response to the scaffolds *in vitro* will be carried out.

## 5. Conclusion

Three kinds of PLGA scaffolds with different pore architectures were successfully fabricated to assess their repair ability for eyelid defects in a rabbit model. The axially aligned scaffold presented bionic structure mechanical property. The axially aligned scaffolds could well support and guide cellular activities *in vitro* and *in vivo*. The *in vivo* infiltration of reparative tissue into the scaffolds could be significantly enhanced in the axially aligned scaffolds, contributing to better repair effects compared with those of the random scaffold. Structural and functional repair of eyelid was realized with fibrous connective tissue integration in the two types of axially aligned scaffold groups，O-PLGA and AZM@O-PLGA scaffolds at 8 weeks post-surgery. Overall, a faster degradation with matched integration rate was found in the axially aligned scaffold group. By loading Azithromycin, the AZM@O-PLGA scaffolds showed a better appearance of eyelid with milder scar. In conclusion, the axially aligned PLGA scaffolds, especially the ones loaded with Azithromycin, provide a promising alternative for eyelid tarsal plate substitutes, and possess greater potential to be translated into medical devices for applications in the future.

## Data availability statement

The original contributions presented in the study are included in the article/Supplementary material, further inquiries can be directed to the corresponding author.

## Ethics statement

The animal study was reviewed and approved by the Animal Ethics Committee of Hangzhou Medical College.

## Author contributions

JY designed this study and provided technical support. PX and PC performed the experiments and wrote the manuscript. YS and JC helped cell culture and animal experiments. QG and HW helped revised manuscript. All authors contributed to the article and approved the submitted version.

## Funding

This study is financially supported by the National Natural Science Foundation of China (82101080, 82000857) and the Natural Science Foundation of Zhejiang province (LQ22H120001).

## Conflict of interest

The authors declare that the research was conducted in the absence of any commercial or financial relationships that could be construed as a potential conflict of interest.

## Publisher’s note

All claims expressed in this article are solely those of the authors and do not necessarily represent those of their affiliated organizations, or those of the publisher, the editors and the reviewers. Any product that may be evaluated in this article, or claim that may be made by its manufacturer, is not guaranteed or endorsed by the publisher.
