# Supplementary material for: Azithromycin-carrying and microtubule-orientated biomimetic poly (lactic-co-glycolic acid) scaffolds for eyelid reconstruction
Source: Front Med (Lausanne). 2023 May 16;10:1129606. doi: 10.3389/fmed.2023.1129606 (PMC10227510; doi:10.3389/fmed.2023.1129606)
Supplement: Supplementary file 1 [file Data_Sheet_1.docx]

**Azithromycin-carrying and microtubule-orientated biomimetic poly(lactic-co-**

**glycolic acid) scaffolds for eyelid reconstruction**

**Xu peifang1#, Chen pengjie1#, Gao qi1, Sun yiming1, Cao Jing1，Ye juan1***

1Department of Ophthalmology, The Second Affiliated Hospital of Zhejiang University, College of Medicine, Hangzhou, Zhejiang, China#

#These two authors contributed equally to this article.

* Corresponding author: Ye juan, yejuan@zju.edu.cn

**Supplementary information**


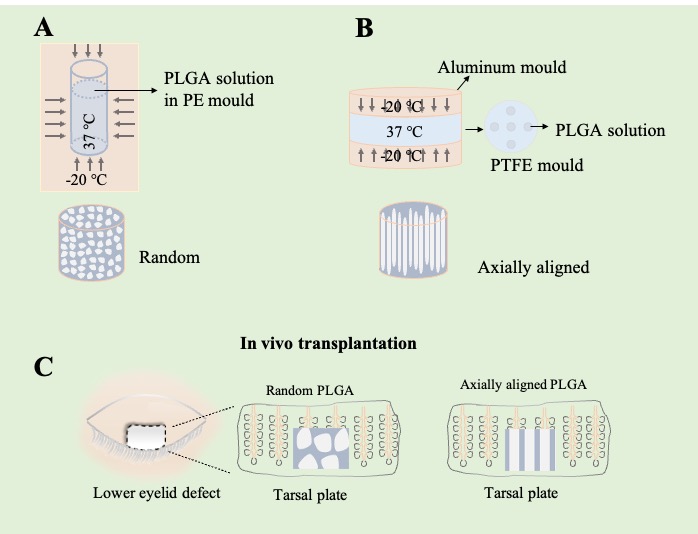


Fig.S1

Schematic illustration of the architectures of PLGA scaffolds with random pores and axially aligned pores, and the mechanism of their implantation into eyelid defects in vivo.

Fig. S2 Azim solution standard curve.


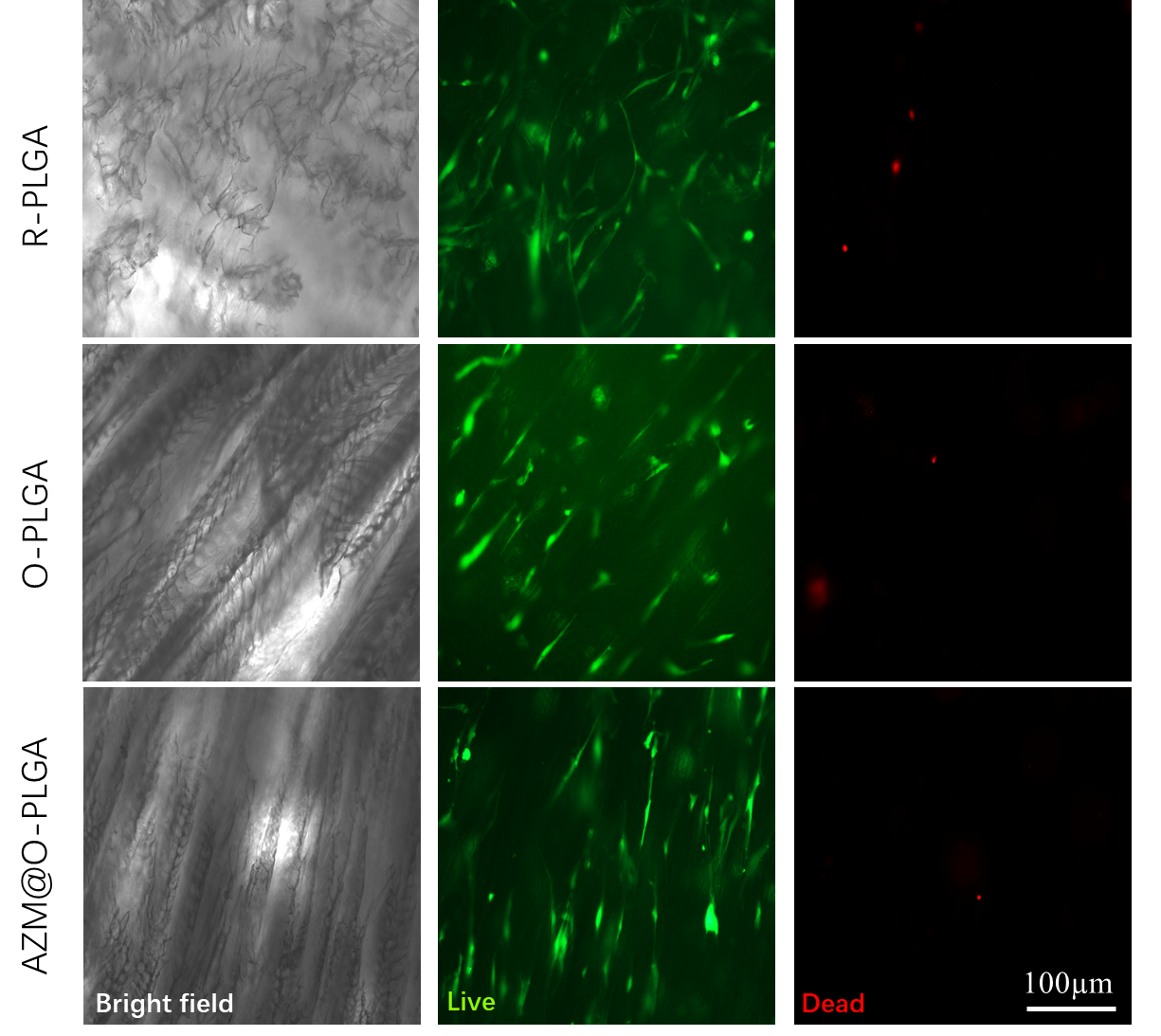


Fig.S3

Live/dead fluorescence results showing the viability of HDFs inside the three scaffolds after 2 days culture in vitro. Live cells are shown in green (calcein AM) while dead cells are shown in red (PI), respectively.


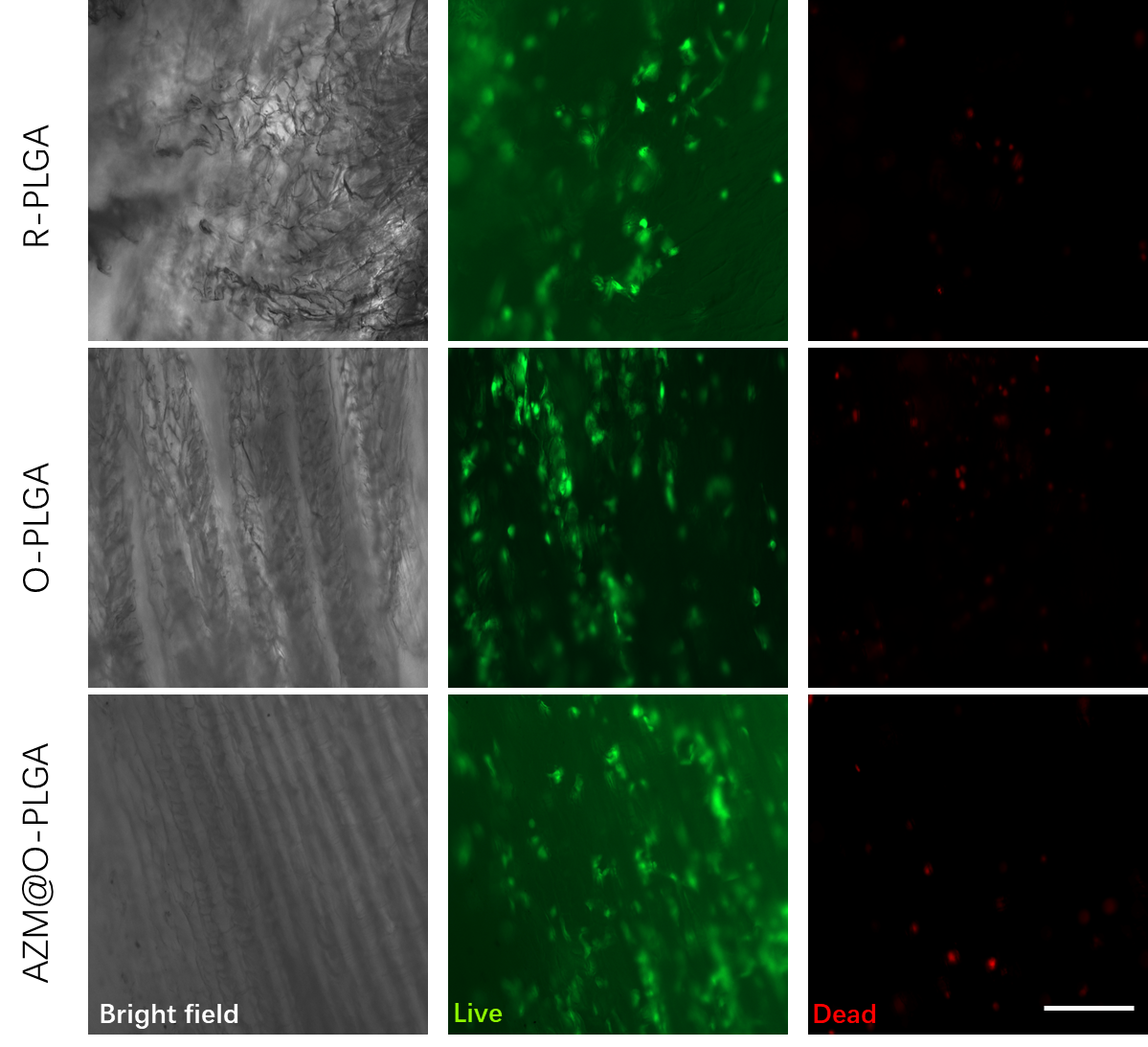


Fig.S4

Live/dead fluorescence results showing the viability of rMGCs inside the three scaffolds after 2 days culture in vitro. Live cells are shown in green (calcein AM) while dead cells are shown in red (PI), respectively.


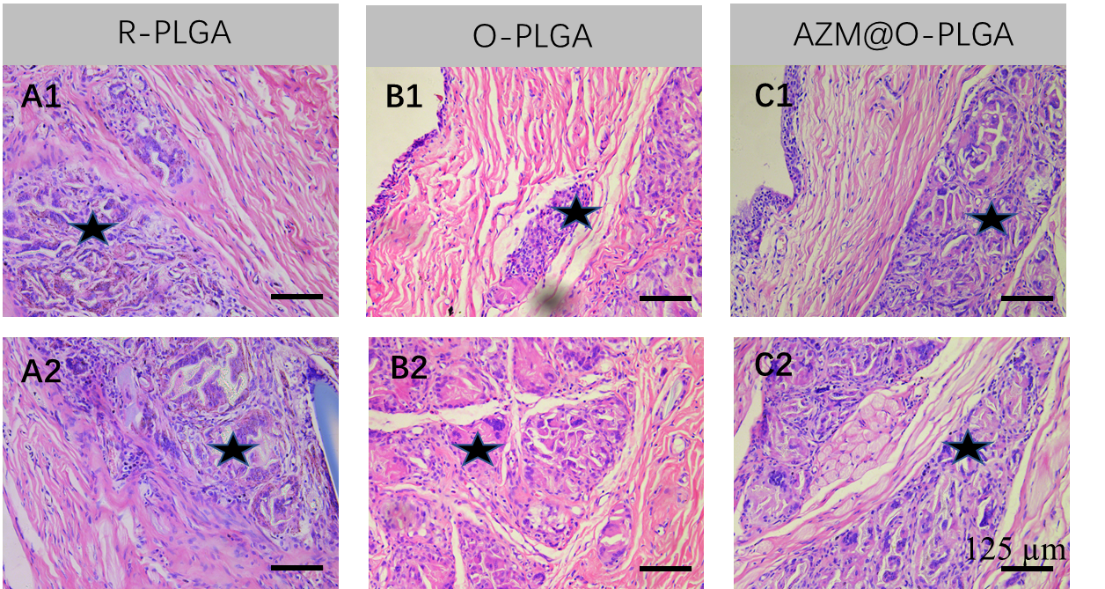


Figure S5. H&E staining of implanting (A) R-PLGA, (B) O-PLGA and (C) AZM@O-PLGA scaffolds for 8 w, respectively. Asterisks point to the remaining scaffolds.


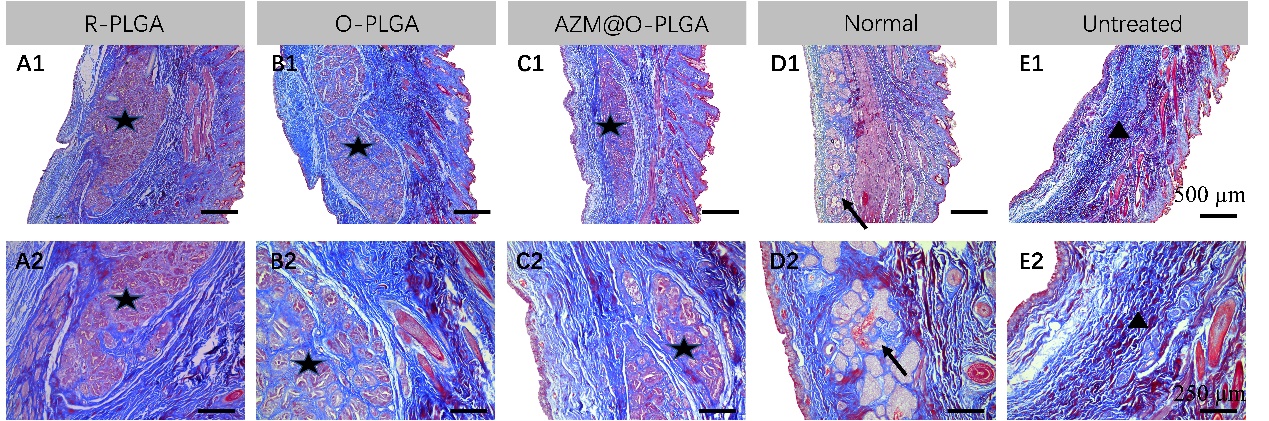


Figure S7. Masson staining of the repaired eyelid implanted by (A) R-PLGA, (B) O-PLGA and (C) AZM@O-PLGA scaffolds for 8 w, as well as (D) normal eyelid tissues and (E) untreated eyelid. Asterisks represent scaffolds. Black arrows point to the normal meibomian glands. Triangles point to the defected site.

**Tables**

Table S1 Primers used for quantitative real-time PCR.

| **Primer** | **Segment** | **Sequence** |
| --- | --- | --- |
| β-actin | Forward (5’-3’) | TGCTATGTTGCCCTAGACTTCG |
|  | Reverse (3’-5’) | GTTGGCATAGAGGTCTTTACGG |
| Krt14 | Forward (5’-3’) | TCAAAGACTACAGCCCCTACTTCA |
|  | Reverse (3’-5’) | GCGTAGGCCATTGATGTCAGA |
| Krt5 | Forward (5’-3’) | GTCAAGAAGCAGTGTGCCAA |
|  | Reverse (3’-5’) | AGCCAGAAGAGAGGCTGTTAG |
| P63 | Forward (5’-3’) | CAAAGAACGGCGATGGTACG |
|  | Reverse (3’-5’) | CGGCCTCTCACTGGTAGGTA |
| SOX9 | Forward (5’-3’) | CAAGAACAAGCCACACGTCA |
|  | Reverse (3’-5’) | AGGGTCTCTTCTCGCTCTCG |
| PPAR-γ | Forward (5’-3’) | CGGAAGCCCTTTGGTGACTT |
|  | Reverse (3’-5’) | CTCGATGGGCTTCACGTTCA |
